# The effect of robot-assisted versus standard training on motor function following subacute rehabilitation after ischemic stroke – protocol for a randomised controlled trial nested in a prospective cohort (RoboRehab)

**DOI:** 10.1186/s12883-024-03734-9

**Published:** 2024-07-04

**Authors:** Jon Skovgaard Jensen, Anders Stengaard Sørensen, Christina Kruuse, Helle Hvilsted Nielsen, Cecilie Dollerup Skov, Henrik Boye Jensen, Marion S. Buckwalter, Jens Bojsen-Møller, Kate Lykke Lambertsen, Anders Holsgaard-Larsen

**Affiliations:** 1 Orthopaedic Research Unit, Department of Clinical Research, University of Southern Denmark, Odense, Denmark; 2 Department of Orthopaedics and Traumatoloy, Odense University Hospital, J.B. Winsløwsvej 4, Odense, 5000 Denmark; 3 SDU UAS Center, The Maersk Mc-Kinney Moller Institute, University of Southern Denmark, Odense, Denmark; 4 Department of Brain and Spinal Cord Injuries, Neuroscience Centre, Copenhagen University Hospital - Rigshospitalet, Copenhagen, Denmark; 5 Neurobiology Research Unit, Department of Molecular Medicine, University of Southern Denmark, Odense, Denmark; 6 Department of Neurology, OUH, and BRIDGE - Brain Research Inter Disciplinary Guided Excellence, Department of Clinical Research, University of Southern Denmark, Odense, Denmark; 7 Brain and Nerve Diseases, Department of Regional Health Research, Lillebaelt Hospital, University of Southern Denmark, Kolding, Denmark; 8 Department of Neurology and Neurological Sciences, and, Department of Neurosurgery, Stanford School of Medicine, Stanford, CA USA; 9 Research Unit of Muscle Physiology and Biomechanics, Department of Sport Science and Clinical Biomechanics, University of Southern Denmark, Odense, Denmark

**Keywords:** Stroke, Body weight unloading, Gait training, Functional training, Neurorehabilitation, Motor learning, Neuroplasticity

## Abstract

**Background:**

Body weight unloaded treadmill training has shown limited efficacy in further improving functional capacity after subacute rehabilitation of ischemic stroke patients. Dynamic robot assisted bodyweight unloading is a novel technology that may provide superior training stimuli and continued functional improvements in individuals with residual impairments in the chronic phase after the ischemic insult. The aim of the present study is to investigate the effect of dynamic robot-assisted versus standard training, initiated 6 months post-stroke, on motor function, physical function, fatigue, and quality of life in stroke-affected individuals still suffering from moderate-to-severe disabilities after subacute rehabilitation.

**Methods:**

Stroke-affected individuals with moderate to severe disabilities will be recruited into a prospective cohort with measurements at 3-, 6-, 12- and 18-months post-stroke. A randomised controlled trial (RCT) will be nested in the prospective cohort with measurements pre-intervention (Pre), post-intervention (Post) and at follow-up 6 months following post-intervention testing. The present RCT will be conducted as a multicentre parallel-group superiority of intervention study with assessor-blinding and a stratified block randomisation design. Following pre-intervention testing, participants in the RCT study will be randomised into robot-assisted training (intervention) or standard training (active control). Participants in both groups will train 1:1 with a physiotherapist two times a week for 6 months (groups are matched for time allocated to training). The primary outcome is the between-group difference in change score of Fugl-Meyer Lower Extremity Assessment from pre-post intervention on the intention-to-treat population. A per-protocol analysis will be conducted analysing the differences in change scores of the participants demonstrating acceptable adherence. A priori sample size calculation allowing the detection of the minimally clinically important between-group difference of 6 points in the primary outcome (standard deviation 6 point, α = 5% and β = 80%) resulted in 34 study participants. Allowing for dropout the study will include 40 participants in total.

**Discussion:**

For stroke-affected individuals still suffering from moderate to severe disabilities following subacute standard rehabilitation, training interventions based on dynamic robot-assisted body weight unloading may facilitate an appropriate intensity, volume and task-specificity in training leading to superior functional recovery compared to training without the use of body weight unloading.

**Trial Registration:**

ClinicalTrials.gov. NCT06273475.

Trial status: Recruiting.

Trial identifier: NCT06273475.

Registry name: ClinicalTrials.gov.

Date of registration on ClinicalTrials.gov: 22/02/2024.

**Supplementary Information:**

The online version contains supplementary material available at 10.1186/s12883-024-03734-9.

## Background

Ischemic stroke is an acute focal injury to the central nervous system (CNS) caused by a disturbance in the blood circulation of the brain leading to cerebral infarction. The increased neuroplasticity observed in the initial weeks after ischemic stroke has been marked as a critical period for subacute rehabilitation [1, 2]. However, in the chronic phase, 6 months post-stroke, individuals may display residual impairments in motor, muscle, physical, and cognitive function in addition to depression, mental and exertional fatigue, reduced aerobic capacity, and reduced quality of life [3–8]. Several rehabilitation strategies have been employed for stroke-affected individuals in the chronic phase [9, 10], however, most studies have included individuals with mild/moderate disability and not severely impaired individuals [4, 9]. Hence, intervention studies are needed to highlight the efficacy of chronic-phase stroke-rehabilitation in individuals with moderate to severe disability despite subacute rehabilitation.

Body weight unloading (BWU) has been suggested as a training method for people with neurological disorders who suffer from severe limitations in walking ability [11]. BWU is the application of a vertical upwards force on the body centre of mass, which reduces the kinetic requirements of locomotion [12]. BWU may allow severely impaired stroke-affected individuals, who are unable to support their own body weight against gravity, to achieve an appropriate and adjustable intensity and volume for recovery of physical function. Furthermore, this method alleviates the therapist of supporting the individual against gravity during weightbearing exercises enabling a more dedicated focus on the quality of the exercise [11, 12]. However, there is limited evidence to support the efficacy of BWU in chronic stroke rehabilitation [9, 10]. Motor learning is a central part of neurorehabilitation interventions [12, 13] due to the associated neural adaptations [14–17]. Basic science studies within motor learning has led to important principles for neurorehabilitation such as active mental engagement, task-specificity, appropriate intensity, volume, and assistance and continuously providing variable and goal-oriented challenges [15, 18–20]. However, earlier studies on BWU exhibit differences, particularly within intensity and volume of training [9]. Furthermore, studies have differed in choice of control group (BWU gait training versus overground gait training, conventional physical therapy, or no intervention), and inclusion criteria (lack of studies on non-ambulatory individuals) [9]. Taken together, these factors may have contributed to the limited efficacy shown for BWU in chronic stroke rehabilitation.

In the present study, robot-based BWU technology yielding a dynamic type of unloading [21] will be employed. The use of this technology has previously been shown to be feasible and safe in healthy adults [22]. Additionally, this dynamic BWU is not limited to facilitating treadmill gait training but can also be used to facilitate overground walking, stair negotiation, and functional task-specific training such as sit-to-stand, squat, and lunges. Dynamic unloading provides a modulated continuously accommodating force that is highly responsive to the movements of the unloaded individual, and this may be superior to no BWU by yielding a more appropriate intensity and volume in training. Furthermore, the presently employed dynamic BWU has been shown to preserve biomechanical gait patterns of healthy individuals [23] and it may be that the current type of dynamic BWU facilitates greater task-specificity in training compared to training without BWU.

The aim of the present study is to investigate the effect of a 6-month robot-assisted dynamic BWU intervention versus standard training, initiated 6–18 months post-stroke, on motor function, physical function, fatigue, and quality of life in a moderately-to-severely impaired stroke population. The main hypothesis of the study is that robot-assisted dynamic BWU training has a superior effect in comparison to standard training on motor function (Fugl-Meyer Lower Extremity Assessment; primary outcome), physical function, fatigue, and quality of life in stroke-affected individuals, still suffering from moderate to severe disabilities after standard subacute rehabilitation.

## Methods

### Experimental Protocol

#### Study design

The present project comprises a randomised controlled trial (RCT) nested in a prospective cohort. The RCT will be conducted as a multicentre parallel-group superiority of intervention study with assessor-blinding. Stroke-affected individuals will be included in a prospective cohort at 3 months post-stroke until 18 months post-stroke with the aim of investigating recovery according to standard rehabilitation i.e. “usual care”. Eligible individuals (those with persistent moderate to severe disability) will be recruited to the RCT-study from the prospective cohort and/or municipal rehabilitation at 6–18 months post-stroke. Individuals not eligible for and/or not interested in the RCT-study will continue their participation in the prospective cohort or municipal rehabilitation.

**Fig. 1:**
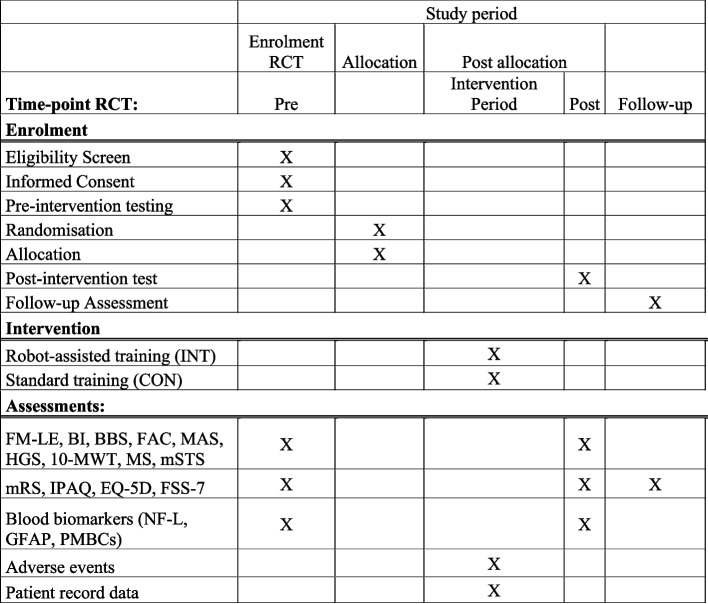
SPIRIT Figure. Template of content for the schedule of enrolment interventions, and assessments. Spirit Figure for the randomised controlled trial. Abbreviations: FM-LE: Fugl-Meyer Lower Extremity Assessment of Motor Function; BI: Barthel Index; BBS: Berg Balance Scale; FAC: Functional Ambulation Category; MAS: Modified Ashworth Scale; HGS: Hand-grip strength; 10-MWT: 10-meter walking test; MS: Muscle Strength; mSTS: modified sit-to-stand; mRS: modified Rankin Scale; IPAQ: International Physical Activity Questionnaire; EQ-5D: Health related quality of life; FSS-7: Fatigue severity scale 7; NF-L: Neurofilament light chain; GFAP: Glial Fibrillary Acidic Protein; PMBCs: Peripheral Blood Mononuclear Cells

The present protocol was registered at ClinicalTrials.gov (NCT ID: NCT06273475). The primary and certain secondary outcome measurements are based upon recommendation from a core outcome set of measurements [24]. The current protocol follows the SPIRIT recommendations (Standard Protocol Items: Recommendations for Interventional Trails) (see Additional file 1 for the SPIRIT Checklist and Fig. 1 for the SPIRIT Fig. [25]) for the minimum content of a controlled trial protocol, and findings will be reported in accordance with the CONSORT statement [26]. Prior to inclusion, pilot experiments will be conducted on 4–6 stroke-affected individuals.

#### Randomisation

The present RCT will use a stratified block randomisation design. Randomisation will be performed internet-based using REDCap Randomise, allocated 1:1. Allocation sequence will be stratified according to global disability (modified Rankin Scale (mRS) score 3, 4 and 5) in blocks of 2 and 4. Stroke-affected individuals will be randomised to robot-assisted dynamic BWU training (INT) or standard training (CON) following pre-intervention testing. The allocation sequence is provided by a data manager with no clinical involvement in the trial, and it is concealed in a password-protected computer file only accessible to the data manager. Participants will be randomised to the intervention group or the active control group by a member of the research team not involved in outcome assessment or statistical analysis.

#### Blinding

The investigator (JSJ) is blinded towards group allocation, blinded during the primary analysis (c.f. *Statistical Analysis*), and is not involved in outcome assessment. Randomisation is performed after pre-intervention testing, and participants will be asked to refrain from revealing group allocation at post-intervention testing in order to ensure blinding of outcome assessors. The raters conducting pre- and post-intervention testing are blinded towards group allocation. Blinding of study participants and physiotherapists will not be possible due to the nature of the intervention.

#### Inclusion and exclusion criteria

The inclusion criteria are as follows: 1) ischemic stroke, 2) > 3 months post-stroke at inclusion in the prospective cohort 2) chronic state at inclusion in the RCT (6–18 months post-stroke), 3) moderate to severe disability defined as having a mRS score of 3–5 and a Scandinavian Stroke Scale (SSS) leg motor function score of 0–4, 4) subacute rehabilitation should be terminated (subacute phase defined as up till 6 months post-stroke), and 5) approved by referring doctor.

Exclusion criteria are as follows: 1) severe fatigue making study completion improbable, 2) cognitive deficits impeding study participation, 3) inability to walk independently pre-stroke, 4) recurrence of cardiovascular/cerebrovascular accidents, 5) pre-existing neurological diseases or ongoing cancer treatment, 6) previously hospitalised due to stroke, transient ischemic attack (TIA), or subarachnoid haemorrhage (SAH), 7) refusing group allocation, and 8) concurrent participation in another trial potentially interacting with the present trial.

### Recruitment and informed consent

**Fig. 2:**
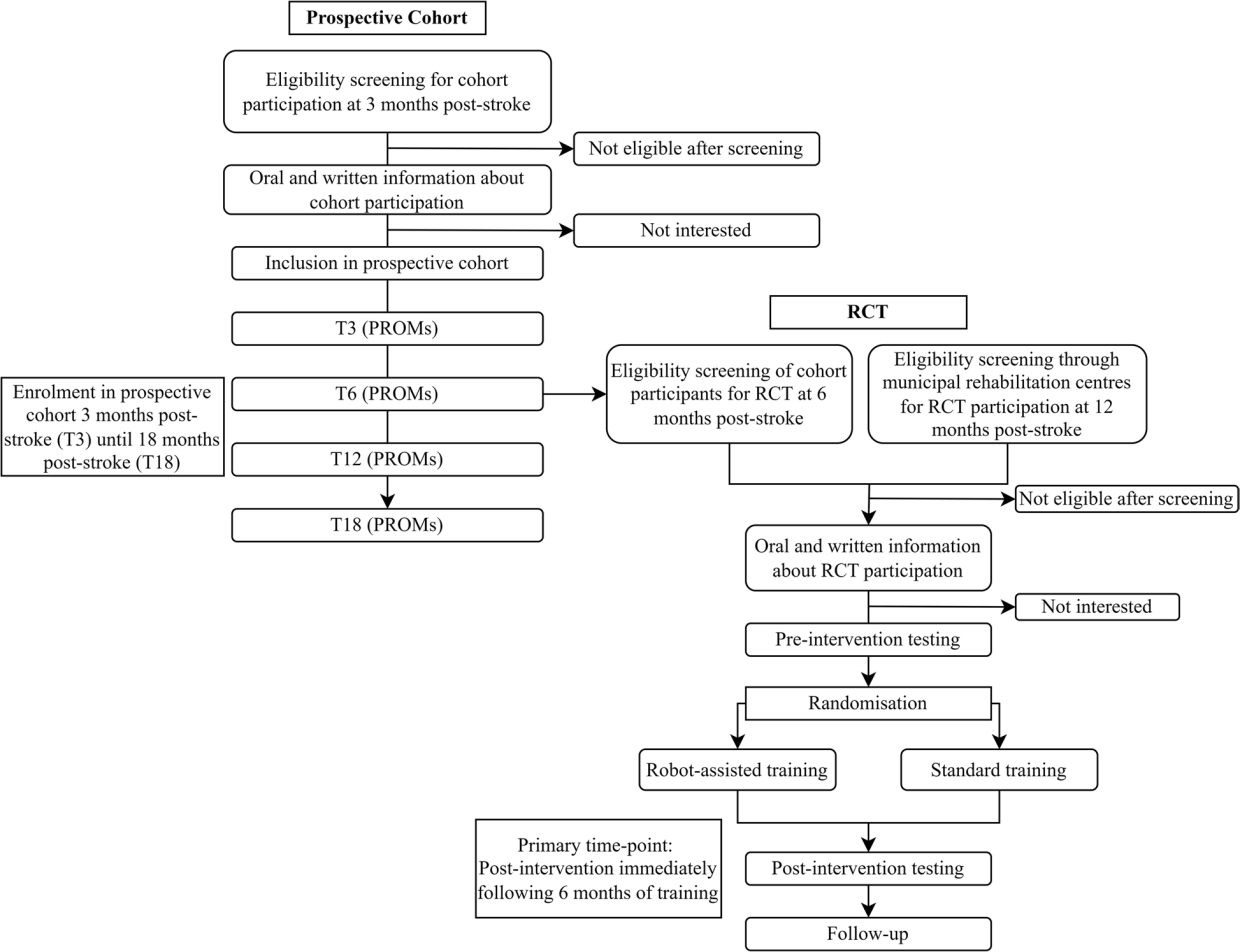
Flow-chart of the study. Abbreviations: PROMs: Patient-reported outcome measurements. Participants not eligible for/interested in RCT participation will continue participation in the prospective cohort

The recruitment and flow of study participants through the present project is highlighted in the patient flow-chart (*c.f. *Fig. 2).

#### Cohort study

Potential study participants will be identified through the 3-months post-stroke standard telephonic clinical examination (mandatory clinical follow-up in Denmark). Patients will be pre-screened ahead of this examination in order to determine eligibility. During the standard telephonic clinical examination, the clinician will provide the eligible patients with a short oral information about the project and ask the person for oral consent allowing the research team to contact the patient to provide in-depth oral information. Patients consenting to later contact will be provided with verbal (telephonic) and written information about the project by a member of the research team. Should the potential participant wish it a family member/friend will be contacted and provided with the same oral and written information. If interested, participants will be asked to give their informed consent (through digital mail) for participation in the prospective cohort including access to patient records.

#### RCT study

Participants in the prospective cohort will be screened 6 months post-stroke to determine RCT-study eligibility, and stroke-affected individuals will be recruited and screened through municipal rehabilitation centres at 12 months post-stroke. If eligible and interested, a member of the research team will provide the participant with written and oral information regarding the project, and the same will be offered to a family member or friend, should the participant wish it. Participants will be offered a physical meeting and will be given at least 24 h to consider before signing the informed consent form. Should the participant wish to participate in the RCT, the informed consent form will be signed on the first day of testing before commencement of any testing procedures.

#### Retention, drop-outs, and discontinuation

Retention will be monitored and promoted by sending systematic reminders to participants regarding questionnaires. Data regarding reason for drop-out or discontinuation is collected and will be reported in the patient flow-chart. Discontinuation may occur 6 months post-stroke for individuals who have regained enough functional capacity to score 1–2 on the mRS or in cases of recurrent cardiovascular/cerebrovascular events (as per the exclusion criteria).

### Intervention

#### Training dosage

The training dosage for both INT and CON will be matched for total hours allocated to training and will consist of 2 sessions per week supervised by a physiotherapist (one-on-one) and last 6 months (48 training sessions in total). Each session will last 60, 75 and 90 min during the first, middle and last two months of the training program, respectively. Thus, INT and CON are matched for training time, but the effective training time or volume load (resistance times repetitions) is not controlled. No study restrictions are imposed on potential regular ‘outside-the-study’ visits to physiotherapists or visits from occupational therapists. Acceptable adherence is defined as a completion of minimum 70% of scheduled training sessions. Moreover, number/percent of completed training sessions and achievement of target intensity/volume will be registered in a training log by the physiotherapist. Detailed intervention descriptions can be found in *Additional File 3*.

#### Training teams

At each trial centre the training team will consist of 1–2 physiotherapists to schedule and conduct the training. Moreover, one external physiotherapist will act as an expert consultant to ensure a comparable intervention between sites and physiotherapists. Regardless of experience, physiotherapists will receive training in intervention delivery. A training brochure compiling possible exercises including progressions/regressions will be developed ahead of and updated during the intervention period. The training teams will have weekly-to-biweekly meetings regarding status/progression of study participants and to ensure physiotherapists are adhering to the prescribed intervention. No formal assessment of competences within intervention delivery will be conducted.

#### Trial sites

Two trial centres are established for the present project (Odense and Copenhagen) to ensure the study meets the target sample size with an adequate flow of participants through the study. The trial centre in Odense is affiliated with Odense University Hospital, and the attached training facility is located at the Department of Sport Science and Clinical Biomechanics (University of Southern Denmark). The trial centre in Copenhagen is affiliated with Herlev and Gentofte Hospital and ‘Rigshospitalet’, and the attached training facility is located at the outpatient clinic at “Bodil Eskesen Centret”. Testing and training will run parallel in Odense and Copenhagen with two different training teams. Experiences/knowledge regarding individualisation/progressions will be shared between trial sites to ensure standardisation of intervention delivery. A technician working on the research project will be responsible for the functionality of the robots.

#### Intervention training

The intervention comprises repeated high-intense task-specific practice of functional movement patterns facilitated by dynamic BWU robotic technology including 1) gait training and 2) functional training and therefore aptly titled “robot-assisted training”. The functional training is designed to strengthen the muscles of the lower extremities and features exercises such as sit-to-stand and stair-walking. A detailed description of the intervention and active control-group training can be found in *Additional File 3.* The presently implemented robot yields a dynamic unloading force applied to the body centre of mass, thereby decreasing the requirements for contractile muscle force production [23]. The BWU robot was developed and custom-built at the University of Southern Denmark using a computer-controlled electric motor combined with force and positions sensors [21]. The robot is mounted in ceiling rails and controls a vertical rope to which the participant is attached (*c.f. Additional File 4: The robot and the harness system*). A static safety harness is also attached thereby ensuring no risk of falling during BWU training. The robot only controls the rope and does not provide mechanical support to guide limb movements. No specific training is conducted for the upper extremities in either INT or CON.

#### Active control group training

The active control group comprises repeated high-intense task-specific practice of functional movement patterns without the use of BWU. CON will consist of “standard training”, which is designed to mimic the current practice of training following subacute rehabilitation. The training program has the same functional goals as INT but is conducted without the assistance of the BWU robot thus only relying on manual support from the physiotherapist, relevant walking aids and a walking track with parallel bars. Participants in CON may perform the same exercises as in INT (e.g., gait training using parallel bars, activation exercises, and functional exercises). Intensity and volume will be regulated by adjusting the amount of physical support and the difficulty of the specific exercise.

### Testing procedure

#### Time-points

In accordance with recommendations for a core outcome set of measurements, the included time-points refer the time since onset of stroke [24]. Thus, time points included in the prospective cohort are 3-, 6-, 12-, and 18-months post-stroke (T3, T6, T12 and T18). Onset of stroke (T0) refers to within 3 days of onset of symptoms [24]. Time points included in the RCT are pre-intervention (Pre) and post-intervention (Post). Subsequently, a follow-up will be conducted 6 months after termination of intervention (Follow-up). Time since stroke will be reported with the study findings for the RCT. The primary time point is immediately after 6 months training (Post) (cf. Table 1).

**Table 1:** Study Outcomes and Time-Points

**Prospective cohort**	**T3**	**T6**	**T12**	**T18**
IPAQ	x	x	x	x
EQ-5D	x	x	x	x
Fatigue Severity Scale 7	x	x	x	x
Modified Rankin Scale	x	x	x	x
Blood Biomarkers	x			
**RCT**		**Pre**	**Post**	**Follow-up**
**Primary outcome:**				
Fugl-Meyer Lower Extremity Assessment		x	x	
**Secondary outcomes:**				
EQ-5D		x	x	x
Fatigue Severity Scale 7		x	x	x
IPAQ		x	x	x
Modified Rankin Scale		x	x	x
Global Rating of Change			x	x
Barthel Index		x	x	
Berg Balance Scale		x	x	
Functional Ambulation Category		x	x	
10-Meter Walking Test		x	x	
Hand-grip Strength		x	x	
**Other outcomes:**				
Muscle strength		x	x	
Modified Sit-to-Stand Test		x	x	
Modified Ashworth Scale		x	x	
Major Depression Inventory		x	x	
Montreal Cognitive Assessment		x	x	
Oxford Cognitive Screen		x	x	
Blood Biomarkers		x	x	

Patient record data from onset of stroke (T0) and from 3 month post-stroke (T3) will be reported. Additionally, data on comorbidities and pharmacological treatment will also be collected at Pre/Post-intervention to assess any changes in these variables during the intervention period and to ensure study participant do not commence pharmacological treatment leading up to the pre- or post-intervention test days

#### Prospective cohort

Study participants in the prospective cohort will receive three electronic questionnaires: The Fatigue Severity Scale-7 (FSS-7), The EQ-5D-5L and the International Physical Activity Questionnaire (IPAQ) (Patient Reported Outcome Measurements; PROMs). A health professional will draw a blood-sample at 3-months post-stroke.

#### RCT-study

Study participants will report for testing on two days both pre- and post-intervention at the training facility affiliated with each trial site. RCT participants will receive the same electronic questionnaires as the prospective cohort and will be asked to answer these ahead of test-days. Participants are encouraged to complete them with a family member/friend. Participants who do not answer the questionnaires at home will be able to do so either on the first day of testing with the outcome assessor or between the first and second day of testing at home with the assistance of a family member.

The two days of testing comprise a series of clinical assessments, performance-based measurements, cognitive tests, a depression questionnaire, and a blood sample. Descriptive characteristics will also be assessed on the first day of testing (age, sex, height, body weight, living arrangements). Estimated testing time for one test day is 2 h. Body weight, time spent training outside the study, adherence and adverse events will be monitored continuously throughout the intervention period.

A medical student or clinical project therapist will be responsible for outcome assessment and will be trained in conducting each test according to a standard operating procedure. Outcome assessors are required to be certified for drawing blood samples and for conducting the Montreal Cognitive Assessment. No formal qualifications are required for the remaining tests, although outcome assessor will be involved in the aforementioned pilot experiments in order to enhance the data quality. No formal experience with the stroke population is required. Outcome assessors will be provided with written material and oral information regarding stroke symptoms including implications for outcome assessment in the present study.

### Patient records

Patient record data will be extracted from participants upon inclusion for the prospective cohort and RCT with the purpose of providing descriptive characteristics/prognostic factors related to the aetiology of the ischemic stroke. The following data will be collected: Onset of stroke (Date), stroke severity at onset (SSS score), type of stroke (ischemic), stroke confirmed on imaging (yes/no), subtype of stroke (large artery atherosclerosis, small vessel occlusion/lacunar, cardioembolic, other determined aetiology, undetermined), location of infarction, hemisphere containing infract (right/left), paretic leg (right/left), acute treatment (thrombolysis, thrombectomy, bolus of acetylsalicylic acid, bolus of clopidogrel or bolus of acetylsalicylic acid and clopidogrel), blood concentrations of leukocytes, neutrophils, lymphocytes, monocytes, thrombocytes, triglycerides, cholesterol, and C-reactive Protein, mRS at 3 months post-stroke, comorbidities (e.g., heart failure, chronic kidney disease, diabetes, atrial fibrillation), and medication (stroke-specific pharmacological treatment from 3 months before study inclusion to post-intervention). Data on comorbidities and pharmacological treatment will be monitored continuously to assess any changes in these variables during the intervention period.

### Outcome measurements

#### Primary outcome

The primary outcome measurement is the between-group difference in the change score of the Fugl-Meyer Lower Extremity Assessment (FM-LE) from pre- to post-intervention on the intention-to treat population. The FM-LE assesses motor function [27] and has been recommended as a part of a core outcome set in stroke rehabilitation studies [24] and is commonly used in stroke research to evaluate motor recovery due to its clinometric properties [28, 29]. The FM-LE assesses reflex activity, voluntary movement, and coordination and velocity of movement through a 0–34 point ordinal scale with each item scored as: 0 = cannot be performed, 1 = partially performed, and 2 = performed completely [27, 30].

#### Secondary outcomes

Secondary outcomes are the between-group differences in change scores from pre- to post-intervention on the intention-to treat population. The outcomes are described below:

##### Modified Rankin Scale

The mRS is a valid and reliable clinical assessment of global disability widely used in clinical settings and recommended as a part of a core outcome set for stroke trials [24, 31]. It’s a mandatory assessment for all stroke patients in Denmark 3 months post stroke. The patient is asked a series of questions to establish the degree of disability or dependence in daily activities with possible scores ranging from 0-6 where 0 = no symptoms and 6 = deceased.

##### Functional Ambulation Classification

The functional ambulation classification is a valid, reliable, and responsive clinical assessment designed to categorise patients according to gait capacity on a 0–5 ordinal scale: 0) Nonfunctional ambulation, 1) Ambulator—Dependent for Physical Assistance Level II, 2) Ambulator—Dependent for Physical Assistance Level I, 3) Ambulator—Dependent for Supervision, 4) Ambulator—Independent Level Surfaces only and 5) Ambulator – Independent [32, 33].

##### Berg Balance Scale

The Berg Balance Scale is a reliable clinical assessment with 14 items each graded from 0 to 4 rating the patient’s ability to maintain positions of varying difficulty and perform specific tasks such as rising from a chair [34].

##### Gait speed

The 10-m walking test is a performance-based measurement to evaluate gait speed. This test is recommended in two forms as a part of a core outcome set; 1) Can the person independently walk 10 m (gait aids permitted) yes/no? and 2) self-selected gait speed on the 10-m test if the person is able to independently walk 10 m [24].

##### Hand grip strength

Hand-grip strength (kg) is a performance-based measurement assessed using a hand-held dynamometer and used in the present study as an indicator for upper limb function [35].

##### Barthel-100

The Barthel-100 Index is a clinical assessment of independence in activities of daily living (ADLs) through observation [36]. The assessment has 10 items (feeding, bathing, grooming, dressing, bowels, bladder, toilet use, transfers, mobility, stairs) and participants may score 0–100 points. This measurement has been recommended as a part of a core outcome set [37].

##### The International Physical Activity Questionnaire

The IPAQ-Short Form is a patient-reported outcome measurement used to measure physical activity over the past week in four different intensity levels, including 1) vigorous activities, 2) moderate activities, 3) walking, and 4) sitting. Higher values equal higher levels of physical activity.

##### EQ-5D-5L

The EQ-5D-5L (European Quality of Life—5 Dimensions) is a validated survey for measuring health-related quality of life [38]. It consists of five dimensions: mobility, self-care, usual activities, pain/discomfort, and anxiety/depression. The outcome is reported on a scale of 1–5 where 1 is no problems and 5 is extreme problems. This measurement has been recommended as a part of a core outcome set [24].

##### Fatigue Severity Scale 7

The FSS-7 is a one-dimensional 7-item patient-reported outcome measurement commonly implemented in stroke trials. Each item is scored from one to seven with higher scores indicating increased fatigue. The FSS-7 was shown to be more valid than the 9-item version of the instrument (FSS-9) [39, 40], and therefore this version of the instrument is implemented.

##### Global Rating of Change

Global Rating of Change is used to assess the participants' overall experience of change from pre- to post-intervention and from post-intervention to follow-up. A seven-point Likert scale is used. Participants will be asked to compare their current overall health to their health 6 months ago with answers ranging from “Much worse”, “Little worse”, “The same”, “Little better”, and “Much better”.

#### Other exploratory outcomes

##### Modified Sit-to-Stand

This test is a modified version of the classic 5-times performance-based chair rise test. Participants will perform 5 sit-to-stand movements as fast as possible but will be provided with body weight unloading to facilitate a more appropriate movement intensity allowing them to finish within 60 second.

##### Muscle strength

Muscle strength is a performance-based measurement assessed in the present study using handheld dynamometry to test the contractile muscle strength of the lower extremities [41].

##### Modified Ashworth Scale

The Modified Ashworth Scale is clinical assessment of spasticity defined as velocity-dependent exaggeration of stretch reflexes. Clinically this is assessed on a 6-point ordinal scale by moving the joints of the participants through full range of motion at a standardised velocity with ratings ranging from 0 (no increase in tone) to 4 (limb rigid in flexion and extension) [42, 43].

##### Major Depression Inventory

The Major Depression Inventory is a dual function questionnaire (diagnostic tool or rating scale). In the present study it is used as a rating scale to indicate the degree of depression on a scale from 0 (no depression) to 50 (maximum depression) [44].

##### Oxford Cognitive Screen

The Oxford Cognitive Screen (OCS) is a stroke-specific cognitive test with 5 domains: Attention and executive function, language, memory, number processing, and praxis [45]. All tasks are scored individually and can be compared against normative data to determine impairments.

##### Montreal Cognitive Assessment

The Montreal Cognitive Assessment (MoCA) is a dementia screening tool commonly used in clinical trials to screen for cognitive deficits following stroke. The MoCA tests the following cognitive domains and provides a total score from 0–30, where 30 indicates best possible cognitive function: visuospatial abilities, executive functions, short-term memory recall, attention, concentration, working memory, language, and orientation to time and space [46].

##### Blood Biomarkers

Venous blood will be drawn from a vein in the cubital fossa by a health professional and collected in 6 mL EDTA tubes or 4 mL vacutainers at 3-months post-stroke (T3) as well as at pre- and post-intervention. Thus, 3 × 18 ml (= 54 ml blood) will be drawn over the course of approximately 9 months if participants are included in both the cohort and RCT (otherwise 36 ml blood in total for RCT and 18 ml for cohort). Samples will be analysed to determine blood concentrations of neurofilament light chain (NF-L) [47, 48] and glial fibrillary acidic protin (GFAP) [49] and changes in peripheral blood mononuclear cells (PBMCs) [50].

### Adverse Events

Resistance training, functional training and gait training (with or without BWU) are well-established and tolerated interventions in the chronic stroke population [3, 12, 28, 51–53], and the health-related benefits for the study participants justify the initiation of the study. The provided training in the intervention and control group will be carried out by physiotherapists who will ensure safety in training. Additionally, when study participants are body weight unloaded, a static safety harness is attached thereby ensuring no risk of falling during BWU training. All training procedures and measurement methods have previously been implemented in stroke-affected individuals or a similar population.

Adverse events (AEs) may include temporary (passing) experiences of local muscle soreness, shortness of breath, fatigue-induced limitations in activities of daily living following training/testing, falls and fractures, which may or may not be indirectly linked to the research intervention [28]. Additionally, drawing blood samples may cause slight discomfort from the needle. All guidelines regarding sterility etc. is followed, whereby minimal discomfort is associated with the procedure, and the risk of infection is very small. In rare cases a small bruise can occur causing local tenderness around the needle-injection site. Should participants regain enough physical function to attempt standing/walking at home, they may be predisposed to an increased risk of falling at home. Therefore, all study participants will be instructed in safety measures if attempting to stand/walk at home. Generally following stroke, there is a risk of recurrent stroke, myocardial infarction, re-hospitalisation and death [28, 54]. All AEs will be collected as data by the physiotherapists in charge of the training intervention (by asking participants and family members) and through patient records, and these will be reported in the main RCT article. The risks associated with participation in the present study are relatively minor in severity. Considering these risks on their own and in relation to the potential advantages of participating, it seems justifiable to initiate the study.

### Data management

Data will be handled responsible in accordance with the General Data Protection Regulation (GDPR) and the rules on protection of personal data from The Danish Data Protection Agency. The Open Patient data Explorative Network (OPEN) in the Region of Southern Denmark will be responsible for data management. The project will be registered at the internal registry at Region of Southern Denmark. Blood samples will be stored in a research-biobank for the purpose of later analysis and will be destroyed following expiration of project approval with the regional ethics committee.

Study data will be managed using REDCap electronic data capture tools. The electronic questionnaires are automatically sent to participants through e-Boks and will automatically appear in the REDCap database. For the RCT, data will be collected on paper, and outcome assessors will be responsible for data entry to the database. The electronic data base is set up with data entry verification to ensure correct values (proper format and within an expected range). Outcome assessors have restricted access to the database in order to prevent them from viewing data from the pre-intervention data collection, and they will not be able to view randomisation codes to maintain blinding. Data entry is performed locally at each trial site into one common database ensuring data is coded the same way.

### Statistical Analysis

#### Primary Analysis

An assessor-blinded intention-to-treat (ITT) analysis will be performed on primary and secondary outcome measures (Primary Analysis). The ITT analysis will employ a two-way analysis of variance (ANOVA) to analyse between-group differences in change-scores from pre- to post-intervention. No interim analysis will be performed. Full analysis data sets will be created with multiple imputation as part of a sensitivity analysis to examine the robustness of the primary analysis.

#### Secondary analyses

Secondary analyses will employ ANOVA to analyse between-group differences in change-scores in the per-protocol dataset (only including participants demonstrating acceptable adherence of > 70%). Secondary analyses will also be performed to evaluate long-term outcome (follow-up 6 months after post-intervention testing) and to evaluate tertiary outcomes not included in the main RCT manuscript.

#### Statistical analysis plan

A separate statistical analysis plan, including potential deviations from trial protocol and their related consequences, will be made publicly available on ClinicalTrials.gov prior to analysis. Unblinding will be performed following the completion of the primary analysis.

#### Sample size calculation

A priori two-sided sample size calculation for two independent groups was performed and resulted in a required sample size of 34 study participants in total. This was based on detecting a minimally clinically important between-group difference (MCID) of 6 points on the FM-LE scale [55] and a standard deviation of 6 points [28, 30, 55] at an α level of 5% and with a statistical power of 80%. Allowing for dropout the aim is to include 40 participants in total.

### Dissemination

Positive, negative, or inconclusive findings will be summed up in manuscripts and submitted at international peer-reviewed scientific journals, presented at international conferences, and published in a PhD dissertation.

## Discussion

This trial is designed to investigate if an intervention based on robot-assisted dynamic BWU yields superior effects on motor function, physical function, fatigue, and quality of life in a chronic stroke population with moderate to severe impairments. This type of intervention may be particularly relevant for this subpopulation within stroke, as BWU-based training may facilitate a more appropriate intensity, volume, and task-specificity thereby leading to superior functional recovery. Non-ambulatory stroke-affected individuals are underrepresented in the scientific literature [4, 9] and thus the trial will not only determine the efficacy of BWU-based interventions for this subpopulation but also contribute to the knowledge on rehabilitation of moderately-to-severely impaired stroke-affected individuals. If the current trial, based upon a controlled and standardized intervention with a high degree of internal validity demonstrates a clear efficacy, the effectiveness of this BWU-training method in a more pragmatic and practice-oriented setting should be investigated. This would be highly relevant to investigate at municipal level rehabilitation, as not all clinics own the costly assistive devices found at higher levels of specialisation in neurorehabilitation. In contrast to such devices, the presently implemented robot is designed with a low-cost principle for ease of broad scale implementation. Taken together, the present trial contributes to the scientific literature on the efficacy of BWU, the rehabilitation of moderately-to-severely impaired stroke-affected individuals, and the findings may guide future studies investigating the effectiveness of BWU in clinical settings.

### Supplementary Information

Additional file 1. SPIRIT Checklist.

Additional file 2. TiDieR Checklist.

Additional file 3. Detailed intervention descriptions.

Additional file 4. Supplementary photos of the robot and harness system.

## Data Availability

The datasets used and/or analysed during the current study are available from the corresponding author upon reasonable request.
