# Supplementary material for: The effect of robot-assisted versus standard training on motor function following subacute rehabilitation after ischemic stroke – protocol for a randomised controlled trial nested in a prospective cohort (RoboRehab)
Source: BMC Neurol. 2024 Jul 4;24:233. doi: 10.1186/s12883-024-03734-9 (PMC11223295; doi:10.1186/s12883-024-03734-9)
Supplement: Supplementary file 1 — Additional file 1. SPIRIT Checklist. [file 12883_2024_3734_MOESM1_ESM.pdf]

SPIRIT

STANDARD PROTOCOL ITEMS: RECOMMENDATIONS FOR INTERVENTIONAL TRIALS

SPIRIT 2013 Checklist: Recommended items to address in a clinical trial protocol and related documents*

Section/item	Item No	Description
**Administrative information**		
Title	1	**Descriptive title identifying the study design, population, interventions, and, if applicable, trial acronym**<br>The Effect of Robot-Assisted Versus Standard Training on Motor Function Following Subacute Rehabilitation after Ischemic Stroke – Protocol for a randomised controlled trial nested in a prospective cohort (RoboRehab).
Trial registration	2a	**Trial identifier and registry name. If not yet registered, name of intended registry**<br>NCT06273475. Registered on 22/02/2024
	2b	**All items from the World Health Organization Trial Registration Data Set**
Protocol version	3	**Date and version identifier**<br>Under "Trial registration"<br>- Registered on 22/02/2024.<br>- Protocol version 6
Funding	4	**Sources and types of financial, material, and other support**<br>Financial:<br>- The A.P. Møller and Chastine Mc-Kinney Møller Foundation (A.P. Møller Fonden),<br>- The Faculty of Health Sciences' Ph.d.-Grant at The University of Southern Denmark<br>- The A.P. Møller Foundation for the Advancement of Medical Science<br>For further information, see *"Funding"* (Page: 23, Line: 503-507).
Roles and responsibilities	5a	**Names, affiliations, and roles of protocol contributors**<br>This information can be found in the section *"Authors' contributions"* (Page: 23, Line: 509-516).
	5b	**Name and contact information for the trial sponsor**<br>The trial sponsor is Odense University Hospital.

	5c	**Role of study sponsor and funders, if any, in study design; collection, management, analysis, and interpretation of data; writing of the report; and the decision to submit the report for publication, including whether they will have ultimate authority over any of these activities**<br>This information can be found in the section *"Funding"* (Page: 23, Line: 503-507).
	5d	**Composition, roles, and responsibilities of the coordinating centre, steering committee, endpoint adjudication committee, data management team, and other individuals or groups overseeing the trial, if applicable (see Item 21a for data monitoring committee)**<br>Not applicable.
**Introduction**		
Background and rationale	6a	**Description of research question and justification for undertaking the trial, including summary of relevant studies (published and unpublished) examining benefits and harms for each intervention**<br>This is provided in the background section of the protocol.
	6b	**Explanation for choice of comparators**<br>The key difference between the intervention group and the active control group in the present randomised controlled trial (RCT) is the use of a robot to facilitate dynamic body weight unloading. As the evidence of the efficacy of BWU in stroke rehabilitation is limited, the present RCT aims to directly compare an intervention with versus without BWU. For this purpose the training in the intervention group and active control group are designed to be similar.
Objectives	7	**Specific objectives or hypotheses**<br>This information can be found in the final paragraph of the background section*"* (Page: 4, Line: 43-50).
Trial design	8	**Description of trial design including type of trial (eg, parallel group, crossover, factorial, single group), allocation ratio, and framework (eg, superiority, equivalence, noninferiority, exploratory)**<br>The RCT is a multicenter randomized (1:1) parallel-group superiority trial, nested in a longitudinal prospective cohort study, with blinded statistical analysis towards group allocation.<br>For further information, see *"Study design"* (Page: 5, Line: 53-62).

Methods: Participants, interventions, and outcomes

Study setting	9	**Description of study settings (eg, community clinic, academic hospital) and list of countries where data will be collected. Reference to where list of study sites can be obtained**<br>Patient recruitment takes place at the following sites:<br>- Odense University Hospital, Department of Orthopedic Surgery, Denmark<br>- Herlev Gentofte Hospital, Department of Brain and Spinal Cord Injuries, Copenhagen, Denmark<br>- Rigshospitalet, Department of Brain and Spinal Cord Injuries, Copenhagen Denmark<br>For further information, see *"Trial Sites"* (Page: 9, Line: 162-173).
Eligibility criteria	10	**Inclusion and exclusion criteria for participants. If applicable, eligibility criteria for study centres and individuals who will perform the interventions (eg, surgeons, psychotherapists)**<br>This information can be found in the section "*Inclusion and exclusion criteria"* (Page 6-7, Line: 92-104).
Interventions	11a	**Interventions for each group with sufficient detail to allow replication, including how and when they will be administered**<br>This information can be found in the section "*Intervention"* (Page: 8-11, line: 137-202) and in Additional File 3: "*Detailed intervention description"*.
	11b	**Criteria for discontinuing or modifying allocated interventions for a given trial participant (eg, drug dose change in response to harms, participant request, or improving/worsening disease)**<br>This information can be found in the section "*Retention, drop-outs, and discontinuation"* (Page: 8, Line: 130-135).
	11c	**Strategies to improve adherence to intervention protocols, and any procedures for monitoring adherence (eg, drug tablet return, laboratory tests)**<br>This information can be found in the section *"Training dosage"* (Page: 8, Line: 138-149).
	11d	**Relevant concomitant care and interventions that are permitted or prohibited during the trial**<br>Per the 8th exclusion criteria "concurrent participation in another trial potentially interacting with the present trial" is prohibited (Page: 6-7, Line: 92-104).<br>As described in the section "*Training dosage"* (Page 8, Line 138-149): "*No study restrictions are imposed on potential regular 'outside-the-study' visits to physiotherapists or visits from occupational therapists"*.

Outcomes	12	**Primary, secondary, and other outcomes, including the specific measurement variable (eg, systolic blood pressure), analysis metric (eg, change from baseline, final value, time to event), method of aggregation (eg, median, proportion), and time point for each outcome. Explanation of the clinical relevance of chosen efficacy and harm outcomes is strongly recommended**<br>The primary outcome, secondary outcomes and other exploratory outcomes are described in the section *"Outcome Measurements"* (Page 13-18, Line 262-384). Additionally, data to be extracted from patient records is described in the section "*Patient records"* (Page 12-13, Line 245-260). Time-points are described in the section "*Time-points"* (Page 11, Line 205-213).<br>An overview of outcome measurements and time-points can be found in Table 1.
Participant timeline	13	**Time schedule of enrolment, interventions (including any run-ins and washouts), assessments, and visits for participants. A schematic diagram is highly recommended (see Figure)**<br>Enrolment procedure is described the section *"Recruitment and informed consent"* (Page 7-8, Line 106-128). See also the patient flow-chart in Figure 1.
Sample size	14	**Estimated number of participants needed to achieve study objectives and how it was determined, including clinical and statistical assumptions supporting any sample size calculations**<br>The sample size calculation can be found under "Sample size calculation" (Page: 21, Line: 451-456).
Recruitment	15	**Strategies for achieving adequate participant enrolment to reach target sample size**<br>As described in the section *"Trial Sites"* (Page 9, Line 162-173)*:* "*Two trial centres are established for the present project (Odense and Copenhagen) to ensure the study meets the target sample size with an adequate flow of participants through the study."*

Methods: Assignment of interventions (for controlled trials)

Allocation:

Sequence generation	16a	**Method of generating the allocation sequence (eg, computer-generated random numbers), and list of any factors for stratification. To reduce predictability of a random sequence, details of any planned restriction (eg, blocking) should be provided in a separate document that is unavailable to those who enrol participants or assign interventions**<br>This information can be found in the section "*Randomisation"* (Page 5-6, Line 72-81).

Allocation concealment mechanism	16b	**Mechanism of implementing the allocation sequence (e.g., central telephone; sequentially numbered, opaque, sealed envelopes), describing any steps to conceal the sequence until interventions are assigned**<br>This information can be found in the section "*Randomisation"* (Page 5-6, Line 72-81).
Implementation	16c	**Who will generate the allocation sequence, who will enrol participants, and who will assign participants to interventions**<br>This information can be found in the section "*Randomisation"* (Page 5-6, Line 72-81).
Blinding (masking)	17a	**Who will be blinded after assignment to interventions (e.g., trial participants, care providers, outcome assessors, data analysts), and how**<br>This information can be found in the section "*Blinding"* (Page 6, Line 83-90).
	17b	**If blinded, circumstances under which unblinding is permissible and procedure for revealing a participant's allocated intervention during the trial**<br>As describe in the section *"Statistical analysis plan"* (Page 20, Line 445-448): *"Unblinding will be performed following the completion of the primary analysis."*

Methods: Data collection, management, and analysis

Data collection methods	18a	**Plans for assessment and collection of the outcome, baseline, and other trial data, including any related processes to promote data quality (e.g., duplicate measurements, training of assessors) and a description of study instruments (e.g., questionnaires, laboratory tests) along with their reliability and validity, if known. Reference to where data collection forms can be found, if not in the protocol**<br>An overview of study outcomes and time-points can be found in Table 1. Further information can be found in the section "*Testing procedure*" (Page 11-12, Line 204-243). References to individual instruments used in data collection can be found in the section "*Outcome measurements*" (Page 13-18, Line 262-383).
	18b	**Plans to promote participant retention and complete follow-up, including a list of any outcome data to be collected for participants who discontinue or deviate from intervention protocols**<br>This information can be found in the section "*Retention, drop-outs, and discontinuation"* (Page: 8, Line: 130-135).
Data management	19	**Plans for data entry, coding, security, and storage, including any related processes to promote data quality (e.g., double data entry; range checks for data values). Reference to where details of data management procedures can be found, if not in the protocol**<br>This information can be found below: *"Data Management"* (Page: 19-20), Line: 411-427).

Statistical methods	20a	**Statistical methods for analysing primary and secondary outcomes. Reference to where other details of the statistical analysis plan can be found, if not in the protocol**<br>This information can be found in the section "*Statistical Analysis"* (Page: 20-21, Line: 429-456).
	20b	**Methods for any additional analyses (e.g., subgroup and adjusted analyses)**<br>This information can be found in the section "*Statistical Analysis"* (Page: 20-21, Line: 429-456).
	20c	**Definition of analysis population relating to protocol non-adherence (e.g., as randomised analysis) and any statistical methods to handle missing data (e.g., multiple imputations)**<br>This information can be found in the section "*Statistical Analysis"* (Page: 20-21, Line: 429-456).
**Methods: Monitoring**		
Data monitoring	21a	**Composition of data monitoring committee (DMC); summary of its role and reporting structure; statement of whether it is independent of the sponsor and competing interests; and reference to where further details about its charter can be found, if not in the protocol. Alternatively, an explanation of why a DMC is not needed**<br>A Data Monitoring Committee (DMC) has not been established because the study is not a test of medical equipment on patients. In accordance with the project aim, the purpose is to investigate the effect of the method of dynamic body weight unloading. In addition, the participants included in the present study are citizens and not patients, as the subacute rehabilitation has been terminated. As also stated in the protocol: "*The Danish National Medical Research Ethics Committee and The Danish Medicines Agency (ref.: 2023112138) declared the project as "not subject to notification*".
	21b	**Description of any interim analyses and stopping guidelines, including who will have access to these interim results and make the final decision to terminate the trial**<br>No interim analyses will be performed.
Harms	22	**Plans for collecting, assessing, reporting, and managing solicited and spontaneously reported adverse events and other unintended effects of trial interventions or trial conduct**<br>Data on adverse events will be collected through self-reported questionnaires, patient records and by asking the study participant and/or family member/friend. Further information is available in the protocol section: "*Adverse Events*" (Page: 18-19, Line: 385-409).

Auditing	23	**Frequency and procedures for auditing trial conduct, if any, and whether the process will be independent of investigators and the sponsor**<br>Not applicable
**Ethics and dissemination**		
Research ethics approval	24	**Plans for seeking research ethics committee/institutional review board (REC/IRB) approval**<br>The study approved the regional ethics committee (Region of Southern Denmark) (Project-ID: S-20230063). The Danish National Medical Research Ethics Committee and The Danish Medicines Agency (ref.: 2023112138) declared the project as "not subject to notification".
Protocol amendments	25	**Plans for communicating necessary protocol modifications (e.g., changes to eligibility criteria, outcomes, analyses) to relevant parties (e.g., investigators, REC/IRBs, trial participants, trial registries, journals, regulators)**<br>Protocol amendments will be communicated through the statistical analysis plan, which will be made publicly available the trial registry (ClinicalTrials.Gov) prior to analysis.
Consent or assent	26a	**Who will obtain informed consent or assent from potential trial participants or authorised surrogates, and how (see Item 32)**<br>Eligible patients will receive verbal and written information about the conditions of the trial and sign a standardised consent form. A member of the research team will provide information and obtain informed consent.
	26b	**Additional consent provisions for collection and use of participant data and biological specimens in ancillary studies, if applicable**<br>Not applicable
Confidentiality	27	**How personal information about potential and enrolled participants will be collected, shared, and maintained to protect confidentiality before, during, and after the trial**<br>The data is stored pseudonymised with a user ID on an online electronic data capture website (REDCap). Data will be analysed through a secure online statistical analysis environment provided by the data manager "The Open Patient data Explorative Network – Region of Southern Denmark". All datasets will be password-protected. Confidentiality is ensured by removing identifying participant information ahead of sharing data with project team members.
Declaration of interests	28	**Financial and other competing interests for principal investigators for the overall trial and each study site**<br>The authors declare that they have no competing interests.
Access to data	29	**Statement of who will have access to the final trial dataset and disclosure of contractual agreements that limit such access for investigators**<br>The datasets used and/or analysed during the current study are available from the corresponding author upon reasonable request.

Ancillary and post-trial care	30	**Provisions, if any, for ancillary and post-trial care and for compensation to those who suffer harm from trial participation**<br>Not applicable
Dissemination policy	31a	**Plans for investigators and sponsor to communicate trial results to participants, healthcare professionals, the public, and other relevant groups (e.g., via publication, reporting in results databases, or other data-sharing arrangements), including any publication restrictions**<br>The study will be submitted for publication regardless of negative, positive, or inconclusive results.
	31b	**Authorship eligibility guidelines and any intended use of professional writers**<br>The four ICJME criteria has been followed.
	31c	**Plans, if any, for granting public access to the complete protocol, participant-level dataset, and statistical code**<br>The datasets used and/or analysed during the current study are available from the corresponding author upon reasonable request.
Appendices		
Informed consent materials	32	**Model consent form and other related documentation given to participants and authorised surrogates**<br>Before inclusion, eligible patients will receive verbal and written information about the conditions of the trial and sign a standardised consent form.
Biological specimens	33	**Plans for collection, laboratory evaluation, and storage of biological specimens for genetic or molecular analysis in the current trial and future use in ancillary studies, if applicable**<br>Blood biomarkers are included as outcome measurements (see "*Blood Biomarkers*" Page 18, Line 376-383) and the data management procedure for these blood samples is describe in the protocol (see "*Data Management*" Page 19-20, Line 411-427).

*It is strongly recommended that this checklist be read in conjunction with the SPIRIT 2013 Explanation & Elaboration for necessary clarification on the items. Amendments to the protocol should be tracked and dated. The SPIRIT Group copyrights the SPIRIT checklist under the Creative Commons "Attribution-NonCommercial-NoDerivs 3.0 Unported" license.
