# Supplementary material for: The effect of robot-assisted versus standard training on motor function following subacute rehabilitation after ischemic stroke – protocol for a randomised controlled trial nested in a prospective cohort (RoboRehab)
Source: BMC Neurol. 2024 Jul 4;24:233. doi: 10.1186/s12883-024-03734-9 (PMC11223295; doi:10.1186/s12883-024-03734-9)
Supplement: Supplementary file 3 — Additional file 3. Detailed intervention descriptions. [file 12883_2024_3734_MOESM3_ESM.pdf]

Intervention description: Intervention and active control group training:

Theoretical foundation

The theoretic foundation of the training programs in INT and CON stems from principles of motor learning (4). Thus, emphasis is on repeated high-intense task-specific practice of functional movement patterns, where the participant is actively engaged cognitively to promote actual motor skill-learning, as such parameters are important drivers of use-dependent motor learning (1–4). The functional goals in both INT and CON are the same, focusing on improvements in walking distance, walking speed, and movement quality (normalisation of movement kinematics and spatiotemporal parameters and reduction in asymmetry between paretic and non-paretic limbs). Movement quality will not be overly emphasised during training sessions, as motor learning may be reduced when movement kinematics are overly guided (2). Therefore, the present study allows an assist-as-needed approach to promote motor learning. Ankle foot orthoses and knee braces are also allowed to minimise any orthopaedic concerns. The training team will establish individual goals and will gradually increase the demands for muscle strength/power, coordination, and postural stability as BWU/manual support decreases.

Gait training

The gait training will be conducted both overground and on treadmill and the initial level of body weight unloading/manual support will be adjusted to allow 5-30 minutes of active gait training. Progression through the training program will continuously be monitored by the training team and participants will progress in walking speed and/or (decrease) in BWU/manual support according to improvements in physical function. Thus, when the physiotherapist determines that the participants has achieved an adequate walking distance and speed with minimal exertion while maintaining good sensorimotor control and coordination (visual assessment).

Functional training

The included functional training targets lower-extremity motor, muscle, and physical function and is thus designed to strengthen the muscles of the lower extremities. BWU and manual support will be used to adjust training intensity and volume. Functional training comprises activation exercises of isolated limbs and functional exercises. The activation exercises are individualised single-joint movements based on the study participants' specific impairments (flexion/extension in knee, hip, and ankle, with the possibility of adding limb loading/unloading to the movement to achieve the appropriate intensity). The functional exercises reflecting ADLs include sit-to-stand, step-ups/step-downs, and stair-walking. Functional exercises may be varied to create regressions and progressions thereby adjusting the neuromuscular intensity and demands for coordination of a task to alter the difficulty. The target volume will be 2-3 sets of 4-15 repetitions. Progression through the training program will continuously be monitored by the physiotherapist and participants will progress according to physical function. Thus, when the participant has shown adequate improvement in the number of performed reps/sets in a given exercise, as evaluated by the physiotherapist, the intensity may be increased through a decrease in BWU/manual support or a progression in task-difficulty. Supplementary exercises may also be included as appropriate for individual participants (e.g., trunk stabilisation and balance exercises) to aid transition to training with less/no BWU or less/no manual support from physiotherapists.

References:

1. Hornby TG, Straube DS, Kinnaird CR, Holleran CL, Echauz AJ, Rodriguez KS, et al. Importance of specificity, amount, and intensity of locomotor training to improve ambulatory function in patients poststroke. Top Stroke Rehabil. 2011;18(4):293–307.

2. Leech KA, Roemmich RT, Gordon J, Reisman DS, Cherry-Allen KM. Updates in Motor Learning: Implications for Physical Therapist Practice and Education. Phys Ther. 2021 Oct 25;102(1):pzab250.

3. Lohse KR, Lang CE, Boyd LA. Is more better? Using metadata to explore dose-response relationships in stroke rehabilitation. Stroke. 2014 Jul;45(7):2053–8.

4. Nielsen JB, Willerslev-Olsen M, Christiansen L, Lundbye-Jensen J, Lorentzen J. Science-based neurorehabilitation: recommendations for neurorehabilitation from basic science. J Mot Behav. 2015;47(1):7–17.
