# Supplementary figures and images for: The effect of robot-assisted versus standard training on motor function following subacute rehabilitation after ischemic stroke – protocol for a randomised controlled trial nested in a prospective cohort (RoboRehab)
Source: BMC Neurol. 2024 Jul 4;24:233. doi: 10.1186/s12883-024-03734-9 (PMC11223295; doi:10.1186/s12883-024-03734-9)

**Additional File 4: Supplementary photos of the robot and harness system.**

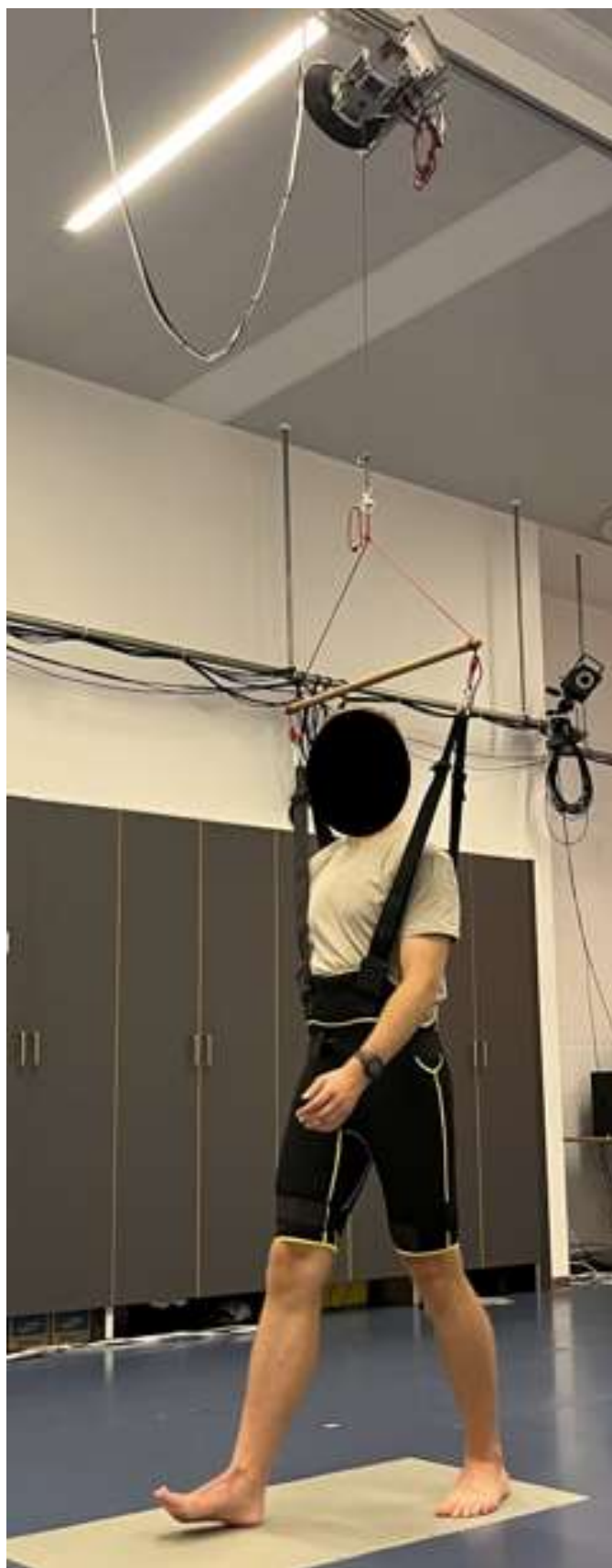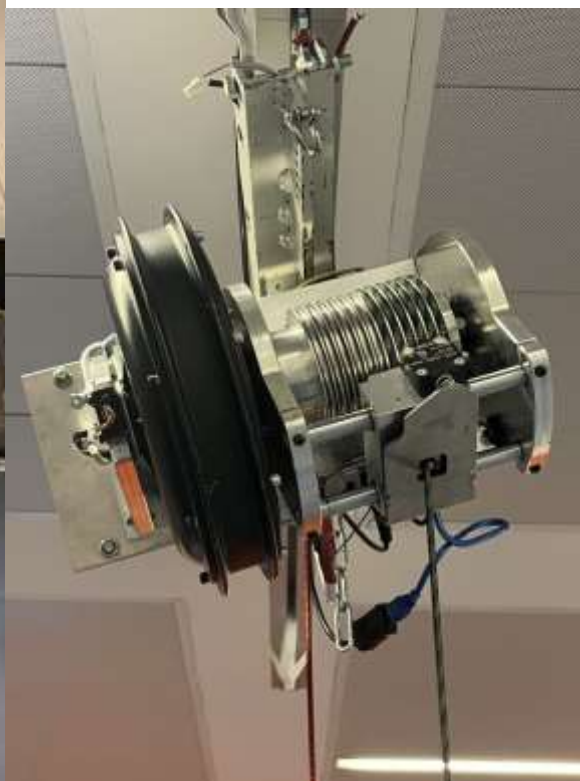

Supplement: Supplementary file 4 — Additional file 4. Supplementary photos of the robot and harness system. [file 12883_2024_3734_MOESM4_ESM.pdf]
